# Experiences of Fathers in Norway Attending an Online Course on Therapeutic Writing After the Death of a Child

**DOI:** 10.1177/10497323231216099

**Published:** 2023-12-01

**Authors:** Olga V. Lehmann, Trine Giving Kalstad, Robert A. Neimeyer

**Affiliations:** 1 Department of Social Sciences, 56627 University of Stavanger, Stavanger, Norway; 2 The Norwegian SIDS and Stillbirth Association, Oslo, Norway; 3 Department of Crisis Psychology, University of Bergen, Bergen, Norway; 4 Department of Psychology, University of Memphis, Memphis, TN, USA; 5 Portland Institute for Loss and Transition, Portland, USA

**Keywords:** therapeutic writing, grief, bereaved fathers, resistance, emotions, meaning-making, poetic representations, journaling, online groups

## Abstract

After the unexpected death of a child, bereaved parents require prompt access to helpful support systems. Online therapeutic writing courses can make such support accessible. Because few studies have included bereaved fathers as participants, we explored the experiences of fathers whose children died unexpectedly and who were part of an online course of therapeutic writing in Norway. We piloted two courses (group 1, six weeks, *n = 9*; group 2, 5 weeks, *n = 5*). We describe our methodological considerations for using poetic representations in qualitative health research and present four poetic representations based on fieldwork notes written by the principal investigator. Then, we triangulate and narratively analyze them together with two collective poems written by participants from each group; excerpts of the writings from two fathers, one per group; evaluation surveys (*n = 4; n = 3*); and, anonymous check-out journaling from the second group (*n = 3*). Resistance was a salient feature of our participants’ grief, and writing enabled them to both be in contact with their emotional world and process difficult emotions as they looked for meaning despite the death of their children. Those who attended the most classes perceived the course as transformative, being part of an ongoing self-exploration, and a possibility to continue and strengthen the bond with their children. Our findings mirror the complexities of the grief experiences of fathers, giving account of their need to find a rhythm to dive into their emotional world, the importance of peer support, and the value of diversifying intervention techniques to meet individual needs and preferences.

There are too few empirically based treatments that acknowledge individual differences in grief, and many of those available treatments are not as helpful for bereaved parents as for other bereaved people (Pelacho-Rios & Bernabe-Valero, 2022). Bereaved parents can be at a heightened risk of developing disorders such as prolonged grief, posttraumatic stress, anxiety, and/or depression (Aoun et al., 2015; Bottomley et al., 2022). Still, about two-thirds perceive that they are not receiving sufficient support as they grieve (Aoun et al., 2015). When the death of a child is unexpected, such as in the case of perinatal loss or SIDS, there are not enough support systems to follow up parents in an often traumatic and challenging process of grief (Corno et al., 2020). Person-centered and individualized support has shown positive effects in helping bereaved parents (Dyregrov et al., 2000), and healthcare professionals mind how factors such as gender, the developmental stage of parents, and the relational nuances with their deceased child can lead to unique journeys of grief integration (Pedraza et al., 2023).

Parents who either feel overwhelmed or feel the need to avoid any expression of grief and mourning tend to struggle the most adapting to life after the death of their children (Barrera, 2007). In terms of couple dynamics, both bereaved parents are said to have shared spaces for affective processing such as providing each other consolation, as well as remembering and maintaining the bonds with the child over time (Bergstraesser, 2015). Some important themes to help parents process and integrate their grief could be acknowledging emotions, resolving unfinished business, and building a form of legacy for the child (Moriconi & Cantero-García, 2022).

Writing is one among many tools that can support individuals and families to attend to their emotional experiences as well as finding motivation and meaning as they nurture the bond with their deceased sons or daughters. Writing can help people embrace the emotional tonalities in their memories and imagination in a way that restores and helps them shape identity with a sense of dignity as they adapt to life course’s challenges (Lehmann & Synnes, 2023). Therapeutic writing promotes affective processing and meaning-making about what makes us suffer—grief, in the case of this article, and thriving in life despite such suffering. Scholars have described the structure and the intend of therapeutic writing techniques in different ways, some of them more focused on exposure to the traumatic experiences (Pennebaker, 1997), and some others, more focused on the creative and dialogical aspects that writing itself can evoke (Neimeyer, 1999, 2012). Either used by the own initiative of the bereaved, as part of low-threshold and/or specialized interventions, writing has proven to have therapeutic effects. The literature in psychology and the humanities provides solid support for the therapeutic effects writing can have when it enhances affective processing and meaning-making of grief, trauma, life transitions, and many other circumstances (Den Elzen et al., 2023; Den Elzen & Lengelle, 2023; Neimeyer, 1999; Pennebaker & Smyth, 2016; Steffen et al., 2022).

In the present study, we target writing among bereaved fathers because very little research on bereavement after the unexpected death of a child includes men as participants (Kersting et al., 2013; Mota et al., 2023). The literature available on the topic centers mostly on parents whose children die due to chronic illnesses such as cancer (Aho, 2009). Meta-analyses emphasize how grief experiences differ between women and men, which leads them to respond differently to interventions (Shut et al., 2001). Among other factors, men can experience a lack of acknowledgment for their needs in grieving, receive less attention from healthcare providers, and experience pressure to support their female partners (Mota et al., 2023; Obst et al., 2020).

Research findings about men’s experiences of grief can be contradictory. Some reports indicate that fathers respond to grief according to masculine gender roles and social norms, coping with their feelings privately and avoiding or silencing their grief while being rational, instrumentally oriented, and emotionally detached (Mota et al., 2023; Williams et al., 2020; Zylla, 2017). Fearing a breakdown, men can also keep busy as a coping strategy, and this motivation competes with wanting or having the need to hold space to relate with their dead child in their daily life (Proulx et al., 2016).

Bereaved parents feel grateful for receiving person-centered psychosocial support where their feelings of sadness or anger are met and validated (Cacciatore et al., 2013). Other feelings, such as shame and guilt, appear to be relevant as well and require attention when providing support (Barr, 2012). Selected findings suggest that bereaved fathers would benefit more from emotionally oriented interventions (Schut et al., 1997), that is, from that which is in opposition to the social representations of their gender. Other perspectives imply that people who cope with grief in more instrumental forms—a more cognitive, action-oriented style often associated with men’s experiences—will seldom participate in individual or group forms of support, especially if such support forms prioritize emotional ventilation over other tools (Doka et al., 2010). In general, men are less likely to seek psychotherapy, and when they do, their dropout rate could be 45% or higher (Seidler et al., 2021).

When it comes to bereavement support groups, some research even suggests that male participants experience an increase in depression symptoms and a decrease in energy and vigorous mood (Maruyama & Atencio, 2008). The way emotions are approached in these interventions, whether these mood changes are temporary or not, and whether they are a part of affective processing of difficult emotions remain to be studied. Innovating in mental health support for men requires a profound understanding of men’s motivations and expectations to develop helpful interventions for them. Whatever the reasons for men to grieve the way they do, researchers and practitioners need to map their needs and wants while integrating perspectives of gender, personality, and culture. Therefore, research and development efforts targeting the exploration of the lived experiences of bereaved parents are crucial to map their needs and preferences and include these in low-threshold and/or specialized support systems. In the present article, we aim at portraying narratives about the emotional world of men in relation to their grief and answer the following questions: what are the lived experiences after the unexpected death of a child that men speak about in an online course on therapeutic writing? How do fathers whose children have died unexpectedly experience being part of such a course on therapeutic writing?

## Methodology

### Study Participants

Nine participants enrolled in the first course; five attended all six sessions, while the other four attended some of the sessions. Five fathers enrolled in the second course, three of them attended all the sessions, one attended some of the group sessions and the individual guidance sessions, and one withdrew from the course, asking if he could instead receive only the emotional first aid from a psychologist. All of them resided in Norway, living in six different cities of the country. The average age of the participants was 35 years. The main causes of death of their children were sudden infant death syndrome (SIDS) or stillbirth (*n* = 9), illness, accident, or not reported (*n* = 5). Aside from two participants who enrolled in courses 14 and 18 years after the death of their children, the average length of the grief of the participants was 15 months. The average age of the children who died by illness and accident was 2.8 years, while for the rest of the participants, the children were born dead or died within a few days after birth.

### Our Writing Courses

For our study, we integrated techniques from diverse theoretical backgrounds in psychotherapy and the humanities, following the premises of pluralistic psychotherapy. Pluralistic psychotherapy emphasizes that human beings have different needs and wants and that providing a diversity of techniques and models will make it more probable to meet these differences of individuals (Cooper & McLeod, 2007). Pluralistic approaches to grief and bereavement have proven to have significant and long-lasting positive effects among their users (Steffen et al., 2022). In Table 1, we present a detailed outline of the course, with a brief description of the psychoeducation and writing practices that we used in both groups. Most of the sessions were alike for both groups. However, as this was a research and development (R&D) project, small modifications were introduced in group 2 in order to attend to some of the feedback from participants in group 1. The general aspects of the course structure and contents are based on a previous study on online writing courses among mothers in Norway (Lehmann et al., 2022).

**Table 1.** Structure and Contents of the Writing Courses for Bereaved Fathers.

Practices repeated every session	Week	Writing practices and psychoeducation sessions per week	
Mindful transition. Guided meditation in group 1. Written practice of awareness where participants describe what they notice in their surroundings and within for group 2 Check-in: Incomplete sentences about what participants feel and do not feel in regard to grief and the course as they start the session Check-out: What have I learnt today about grief/writing/myself?	1	Adapted version of proprioceptive writing (Metcalf & Simon, 2002): Listening to a piece of music and reconstructing experiences about it. Includes reflective questions	
		Psychoeducation about therapeutic writing (Neimeyer, 1999, 2012; Pennebaker & Smyth, 2016)	
		Chapters of our lives: Writing the table of contents of one’s autobiography followed by reflective questions (Neimeyer, 2011)	
	2	Psychoeducation about the dual process model of grief (Stroebe & Schut, 2010)	
		Acrostic with the letters in the name of the child who has died	
	3	Shared reading of Rumi’s poem “The guest house” (Jalāl al-Dīn & Barks, 1996)	
		Psychoeducation on the dialogical self theory (Hermans, 2001)	
		The guests of the inner house: Bringing the wisdom of silent aspects of one’s identity forward through reflective questions (Lehmann et al., 2022)	
		Group 1: Autumn 2022	Group 2: Winter 2023
	4	Psychoeducation about anger and meaning (Frankl, 1990; Pascual Leone & Paivio, 2013).	Psychoeducation about anger, guilt, shame, and meaning (Frankl, 1990; Greenberg & Iwakabe, 2011; Lengelle et al., 2016; Pascual Leone & Paivio, 2013)
		Adaptation of the practice “How would you treat a friend” (Neff & Germer, 2018)	Participants write a dialogue between parts of themselves that are stuck with shame and parts of themselves that bring in compassion
		**Practices that remained the same in both groups**	
		Psychoeducation about compassion and self-compassion (Lehmann, 2018; Lehmann et al., 2022; Neff & Germer, 2018; Shulz & Monin, 2018)	
		Writing about the unfairness beneath anger and what one can possibly learn about it	
		Group 1: Autumn 2022	Group 2: Winter 2023
	5	Psychoeducation about shame and meaning (Greenberg & Iwakabe, 2011; Lengelle et al., 2016)	Modified version of “Introducing a loved one” (Hedke, 2012) where parents introduce their grief in a third person narrative
		Participants write a dialogue between parts of themselves that are stuck with shame and parts of themselves that bring in compassion	
		Modified version of “Introducing a loved one” (Hedke, 2012) where parents introduce their grief in a third person narrative	Same practices as in week 6 for group 1
	6	Second version of chapters of our lives: Writing the table of contents of one’s autobiography followed by reflective questions (Neimeyer, 2011). Reflective questions about it and comparison with draft from week 1	N/A
		Modified version of “correspondence with the deceased” (Neimeyer, 2012). Participants write a letter to their child	
		Writing a collective poem with the prompt “a door is opened”	

Independently of the theoretical premises of the diverse techniques that we used, one of the intentions of these techniques was to promote genuine dialogues between participants and others and parts of themselves. Some of the practices implied explicit dialogue in the form of letters to other course participants, to oneself, and to the deceased (Hedtke, 2012; Neff & Germer, 2018; Neimeyer, 2012). Other activities featured more implicit forms of dialogue within the self, using creative and imaginative tasks, such as sketching the table of contents of one’s biography (Neimeyer, 2011) or writing about grief using poems or songs as prompts (Metcalf & Simon, 2002). Independent of these different practices, writing served as a platform for dialogue between the participants with diverse aspects of their identity, their partners, healthcare professionals, other peers, and their children.

#### Implementing the Writing Courses

We facilitated the first of our writing courses for bereaved fathers in the autumn of 2022 for 6 weeks with 3-hour weekly encounters and a 30-minute session of individual feedback about a chosen text. Given the positive response to the individual sessions, we reorganized the structure of the second course, running it for 5 weeks in the spring of 2023 with 2.5-hour weekly encounters, along with three 30-minute sessions of individual feedback on the chosen text. All participants were offered two free emotional “first aid” sessions with a psychologist to process the difficult emotions that would arise in the course. Both the emotional first aid and individual feedback sessions were popular, even among the participants who dropped out or did not attend all the sessions of the course.

The principal investigator met the study participants via Zoom. Each evening session started with a practice of mindful transition into writing and check-in journaling with incomplete sentences about their feelings at the moment. Psychoeducation about the week’s topic (hope, dialogue, compassion toward shame and anger, self-compassion loving-kindness, and meaning of life, respectively) in relation to grief, emotions, and existential meaning was provided. The writing practices consisted of individual tasks and reflective questions in small groups and in plenary sessions, to foster emotional awareness, affective processing, and meaning-making. Participants were encouraged to decide for themselves how little or how much to write, as well as how little or how much to share with one another. To promote a sense of safety and rhythm for the group dynamics, participants were also reminded that they could speak about their inner processes while writing without reading the exact words they wrote. At the end of each session, the participants journaled based on check-out reflective prompts and received at-home practice.

### Data Collection and Data Analysis

#### Poetic Representations

We crafted four poetic representations based on fieldnotes from the principal investigator while facilitating the two writing courses. These fieldnotes mainly contained summaries of what participants said in the discussions in the plenary group, session by session (11 weeks total). Poetic representations are emotionally compelling syntheses, here in the form of free verse poems written based on data from study participants (Faulkner, 2005, 2009). In qualitative health research, poetic representations are relevant because they can provide insights into the bodily experiences of the suffering of participants (Ohlen, 2003), evoking the multi-voicedness of experiences within a participant and/or across participants. Such syntheses of data also invite a dialogue among participants, the researchers, and audience of the article (Lehmann & Brinkmann, 2021).

One can think about these poetic representations as spoken word poems rather than a classic poetic form. The process of crafting the poetic representations involved the following steps: (a) transcription of the fieldnotes, separating the paragraphs into rows; (b) adding an extra column with codes that summarize the contents of each paragraph; (c) adding an additional column for analytical remarks and themes; (d) refining the codes and finding overarching themes for them; (e) sketching poems to reflect key themes; (f) reading the poems several times and reorganizing the wording and polishing the themes; (g) sending the poems to the coauthors of the article for feedback; (h) sending the poems to the study participants for feedback; (i) incorporating feedback and synthesizing the poems to reduce their length; and (j) refining the aesthetics of the stanzas while keeping faithful to the fieldnote transcriptions.

The final product corresponded to four poetic representations with the following themes: (a) *grief and time* about their experiences of temporality in grief, for some as nuanced by the pandemic; (b) *owning one’s emotions*, regarding the participants’ experience of emotions and emotional processing; (c) *parallel fatherhoods*, about the ambiguous experiences of being fathers of a dead child while opening themselves for new pregnancies; and (d) *grieving as a process*, highlighting the participants’ experiences of grief and meaning-making from a dialogical perspective.

#### Narrative Analysis of the Poetic Representations and the Participant’s Writings

In order to ensure the qualitative validity of our poetic representations, we contrasted them with other forms of data and analyzed all these materials narratively. We followed the premises of narrative psychology, where stories are central to human experience and existence, and researchers are to collect data that resembles how the psychological life of participants is like through the meaning-making processes that give account of it (Josselson & Hammack, 2021). In specific, we paid attention to the key events and the stories these events form, how characters are positioned in the script of the narrative, and the emotional nuances in the narratives (Silver, 2018).

The first two pieces consisted of short poems written collectively in the chat during the last session of each course. The other pieces were two poems written by a participant from each of the two courses and a summary table of the check-in and check-out journaling week by week shared by one of these fathers. In addition, we included the data from anonymous evaluation surveys from both groups, check-out journaling practices from the second group, and an e-mail follow-up with a participant. When possible, we used pseudonyms to refer to the participants and their children. For the data filled out anonymously, we numbered the participants and used the abbreviations “ES1” or “ES2” to specify if the evaluation survey was from the first or second group and then numbered the participants, and we used “CHO” for check-out journaling specifying the session.

#### Reflexivity of the Principal Investigator

The practice of writing fieldnotes when facilitating online groups grounds me. Attending to different emotional rhythms, life stories, and perspectives is humbling; it asks for my listening and of my remembrance. A participant read once a line from an unfinished poem, reiterating the struggle to find words that were faithful to his suffering and to the urge for finding a way back to a life rhythm. The heartbeat of his desire to cherish words continued to resonate in me; he reminded me of the power and responsibility of my own words. As researchers, we often write about the experiences of our participants in rather complicated ways; creative methods in qualitative inquiry are powerful tools to come closer to our participants: they enable us to speak from and to our human condition.

There is much that happens in the context of writing groups that transcends writing itself. It is about the sense of reciprocity being nurtured in these meetings (Lehmann & Brinkmann, 2019). What a participant shares can echo or provide perspective to the experiences of another. They might share the struggle to find words to the ineffable and, while crafting a sense of togetherness, sow the fabric of grief with soft and rough edges. The blanket of their words is a companion as each of them transits the storm of difficult emotions and traumatic memories at their own rhythm. I wrote the four poetic representations as an attempt to interweave the threads of unique life stories of my participants, mirroring the complexities of masculine grief.

Given how tragic it is to be given and withdrawn from a child too quickly, I wanted to voice for these bereaved fathers what I felt to be true in the 11 weeks I attended to their longings. Aware of the interpretative aspect of my recollection of the fieldnotes, I knew my poems would not be sufficient unless their content would be contrasted with actual narratives from the participants themselves because my first and foremost audience is them: I wanted these poems to portray what is to grief from their perspective, not mine. It is therefore that each of the four poetic representations is accompanied by data from surveys, or writings that our participants shared with us, altogether with reflections nourished by academic literature.

### Ethical Considerations

We received the approval number 885234 from the Norwegian Agency for Shared Services in Education and Research (SIKT) to carry out this project, given that our research protocol fulfilled their criteria for data protection. Our project was exempt of approval from the Regional Committee for Medicine and Health Research Ethics (REK) because they considered that our research protocol did not involve major risks for the participants. Our participants also gave us permission to use excerpts of their own writings in this research article.

## Results and Reflections

## Men’s Grief for a Child in Four Stanzas

### First Stanza: On Grief and Time

*Three, eleven months,*

*five years, fourteen?*

*Time isn’t clear.*

*Rushing back to work,*

*new pregnancies,*

*one’s partners’ suffering,*

*a pandemic shutting it all down.*

*No travel, no football, no pub,*

*how unjust, “doing” grief*

*in this unnatural manner.*

*Even for those who had a chance*

*for recreating—trips, drinks, or sports—*

*there was not as much room*

*to speak about men’s grief, as a norm.*

*Melancholia: feeling like I am missing*

*body parts. Grief is physical for us.*

*Isolated, because of this and that—*

*partly because I resist.*

*I have no words, or few words.*

*I let grief freeze,*

*I try moving on with life. It works.*

*Yet, there are reminders,*

*almost daily, that something isn’t*

*as it should be.*

*I struggle when times are good:*

*Am I not going deep into grief?*

*Will the balloon burst?*

*I don’t know if I am including my child*

*enough in my current life.*

*When, and how to grieve?*

*Now, writing: a way of grieving.*

*It’s scary. Perhaps not for me?*

*Echoes of the future, reminiscences.*

*Echoes of the past, my own childhood*

*mirroring current parenthood.*

*Surprised to travel in time,*

*through emotion. I find lessons,*

*things I thought I had processed.*

*I work on them, once again,*

*I have other resources now.*

*On grief and time* is a poem that invites us to reconsider temporality on the light of suffering and love. There are phenomenological aspects of mourning that do not follow a chronological order; they are rather forms of disruptions (Shardlow, 2022). Enabling bereaved fathers to consider nonlinear approaches to temporality seems to allow them to reconstruct their identity narratives more freely (Barak, 2014). During the course, we briefly mentioned that both love and grief were to be measured in terms of the intensity of our feelings, not chronological time. What we did not expect was that this theme would be so recurrent in the descriptions and sharing of our participants, even before the topic was explicitly introduced.

Some perceived that the lock-down during the COVID-19 pandemic affected their perceptions of time, leaving them with several constrains to enroll in conventional activities and/or too much time to grieve. It can be overwhelming for parents to deal with the loss-oriented aspects of grief, and during the COVID-19 pandemic, the disruption of roles, routines, and relationships imposed further challenges for the bereaved to find a time that felt appropriate to grieve (Hooghe et al., 2021). Yet, the topic of the pandemic was not as recurrent as it was the case for the overarching emotional tonalities related to grieving. A sense of ambiguity in terms of how long their grief would last, independent of the number of months or years after the death of their children, was a common experience for all participants. The emotional intensity and the challenges to label and/or process difficult emotions seemed to rule such ambiguity of time, as we explore in the next section.

### Second Stanza: How to Own Emotions

*Paved with resistance,*

*my readiness to grieve,*

*to writing.*

*The hard parts:*

*removing the masks*

*used when telling a grief story.*

*An urge to avoid.*

*Finding words for feelings,*

*letting feelings be.*

*This grief of instances.*

*Grief asks me to say what I mean,*

*truly. Not the headlines,*

*what lies behind.*

*An unexplored forest of emotions.*

*Tension, willingness to be in control.*

*Disbelief for some memories.*

*Then, there is regret.*

*An autopsy that leads nowhere:*

*it was no one’s fault.*

*Why do I keep wanting to find a cause?*

*What if I could have saved us from this tragedy?*

*Unfairness, that’s what I think the most.*

*The despair of wanting*

*to rescue them into life numbs me.*

*I can’t change it. Anger, so much anger.*

*I fear the anger I feel.*

*Anger is a lonely place, to begin with.*

*Then, I notice the unfinished:*

*conversations that didn’t happen,*

*or didn’t go the way they should,*

*with healthcare, family, or friends.*

*Unfinished dialogues with myself,*

*with my partner, with my child.*

*Going into feelings, in a giving way.*

*Now I can see it,*

*how hindered these emotions*

*have remained.*

*There is also the mundane,*

*Honoring other states of mind,*

*the days I want to stay “busier,”*

*the days I need to be more practical.*

*Difficult periods lead to ambivalence.*

*How to own emotions* is a poetic representation that evocates the practice of being in contact with one’s own emotions and the practice of remembrance among our participants. As they grieve, these men attuned with their own emotional rhythm, something that can be challenging and is often experienced in diverse forms of resistance. Such resistance is not only negative: it belongs to grief, and it can give account of instrumental aspects of grief that can potentially lead to constructive patterns of grief integration (Doka et al., 2010; Huang et al., 2023; Schut et al., 1997; Zylla, 2017). Resisting grief might also reflect the pressure that implicit or explicit social norms about gender impose on men (Mota et al., 2023; Zylla, 2017). In addition, our participants, as we will exemplify in the next paragraphs, also needed to develop emotional literacy about difficult emotions such as anger, guilt, and shame (Barr, 2012; Moriconi & Cantero-García, 2022).

#### Integrating Perspectives: The Participants’ Narratives on Emotions

After writing and editing the four poetic representations, we sent them to the course attendants, asking for feedback about whether they felt seen and felt the words conveyed the experiences of the group. One of them replied with the following:

I’m in a period just before the anniversary of the death of my daughter, so I struggle to have enough cognitive capacity while reading these poems. In general, I can say that I liked them and felt seen in a lot of what you wrote. The challenges to express ourselves and find words for feelings was the part that best answered your question about the process in the course, together with the raw emotions that make it difficult to share something. (Steinar, father of Lia)

Steinar’s words suggest what other parents said in the evaluation surveys: that working with emotions and meaning during the online course mattered to our participants, even if they proved difficult. This might indicate, similar to other research findings, that addressing emotions is relevant in grief interventions for bereaved fathers (Schut et al., 1997). Even if the context of our intervention is different, research on parenting courses suggest that working with emotions as parents is painful and confrontative yet meaningful (Ansar et al., 2021). For instance, a participant emphasized that working with emotions involves resistance and tension:

I need a bit of time to connect with my feelings. I need to put a lid on and keep them at a distance to function at work during the day, and this takes a lot of energy, and I need to “warm up a little” or let things in gradually so that I don’t become overwhelmed. (Participant 2, CHO, day 2)

Another participant highlighted in the evaluation survey of being in contact with their core feelings and that he appreciated “being able to write with feelings as a direct departure point, without rationalizing them” (Participant 1, ES1). However, it can also well be that resistance to it might result in feeling overwhelmed and demotivated for some, which also mirrors other perspectives in the literature (Doka et al., 2010).

There are at least two important aspects to consider in these possible explanations: the cultural nuances of emotional literacy and understanding of fatherhood. Studies on emotional literacy indicate that men who express conformity to masculine norms and expectations can have higher symptoms of depression in general compared with other men (Milner et al., 2019). This does not necessarily mean that men feel at a lower intensity but that, when living in accordance with classic views of masculinity, they might strive to deal with difficult emotions covertly, which is something that can both give them a sense of mastery and isolate them or help them avoid emotional processing (Cook, 1988). Even if there are few studies about the ways in which culture affects emotional literacy, regulation, and expression in Norway, preliminary findings have suggested that Norwegian students are less likely to perceive themselves as aware and sensitive to emotional expression and control when compared with American students (Hystad et al., 2010). At the same time, Norway, like other Nordic countries, has promoted a change in the social representations of fatherhood, aspiring to gender equality. This means that, nowadays, Norwegian fathers are expected to engage actively in the care of the child without this threatening their sense of masculinity (Magnunsson et al., 2008). This adds to the complex landscape of the possible experiences of our participants, showing why a focus on culture in further studies would be crucial to gain a better understanding of the needs and wants of bereaved parents.

### Third Stanza: Parallel Fatherhoods

*All we should have lived,*

*together.*

*The dreams of a playful childhood,*

*interrupted*

*by illness, accidents, the unknown.*

*A pregnancy, a birth, a funeral.*

*Duality: being in a maternity ward,*

*families welcoming a new member,*

*and we, saying goodbye.*

*To be a father once more.*

*No one understands … they say,*

*“you must be stronger.”*

*Stronger?*

*It’s difficult as hell to remain enthusiastic*

*as new pregnancies arrive.*

*How to prepare for an upcoming birth?*

*My role as a father of other children,*

*Celebrating a death anniversary*

*just days before a new child is born.*

*Life in its raw nuances.*

*Some find their way*

*to new pregnancies, new children,*

*some don’t.*

*A question sneaks in:*

*will we survive this as a couple?*

*While proud to have become a father,*

*some days are easier than others.*

*Parallel Fatherhoods* is a poetic representation set to convey the ambiguity of affirming our participants’ sense of still being fathers of a child who died too soon, while for some of them this fatherhood merged with the experience of having children again. Writing and reflecting about their grief, these parents were also authoring their identity. The renewal of identity is an important part of the process of integrating grief into everyday life (Den Elzen, 2021; Gillies et al., 2013; Lengelle, 2021). This theme appeared to be as relevant for the fathers as it was for the women who attended online writing courses (Lehmann et al., 2022). As part of this process, parents often imagine the possible lives their children could have lived, feeling confronted with how difficult it can be to continue a bond while having lost them at such an early age (Milman & Rheingold, 2023). The life trajectories of people who undergo transitions such as the death of a beloved one are unique, giving expression to diverse pathways to find existential meaning (Lehmann & Brinkmann, 2021).

#### Integrating Perspectives: The Participants’ Narratives on Fatherhood

In the evaluation survey, some participants shared that loneliness, the challenges of being a father, death, and long-term illness were the most difficult topics to write about. At the same time, there was also a sense of appreciation for being in the course and writing, even if it was difficult. One participant said, “It was nice to see that there were many others in the same unknown territory as me” (ES1), and another added that he learned “that I can withstand my grief—the painful and the good about it” (ES2). In recognizing this, some of our participants affirmed that if writing becomes an opportunity for both meaning-making and affective processing, it is likely to have therapeutic effects (Den Elzen et al., 2023; Lehmann et al., 2022; Neimeyer, 1999; Pennebaker & Smyth, 2016). The following two cases bring forward the theme of identity and fatherhood, along with how challenging it can be to integrate a parallel fatherhood.

##### Artur’s Case

Artur’s poetic prose, *Grieving Nora*, consists of 74 lines and is a philosophical questioning that helped him honor the absurd. Some of his existential inquiries appear once in the text, such as “Suffering? Uneasiness? Insomnia? Longing for closeness?” (line 1) or “Is one one’s own source? With voice, thoughts, meanings, and feelings?” (lines 8–9). Other questions are repeated, evoking the piercing willingness to affirm his daughter’s ephemeral presence, “How are you?” (lines 14, 21, and 30), “who could you have become” (lines 14 and 16), or “were you?” (lines 14, 31, and 44). This is the story of a father who makes a promise: “I will never forget you … even if our time together was so short” (lines 62–64); and the story of a father who asks for a promise: “Never stop visiting me” (line 70), followed by the grand finale “I love you/Nora’s dad, and ‘Noravind’ [Nora’s wind] we follow” (Lines 73 and 74).

In addition, Artur shared (see Table 2) a summary of his check-in and check-out journaling week by week, giving an account of an evolving process of meaning-making. For instance, in the first weeks, there was more self-criticism and caution, while in the last 3 weeks, there was an awareness and processing of emotions, as well as a focus on the continuation of the bond with his daughter.

**Table 2.** Artur’s Check-In and Check-Out Journaling Practices.

Day	Check-in journaling about feelings	Check-out journaling about learning
1	N/A	N/A
2	Neutral, introvert	I have problems sharing with others whom I think have it worse than me.
3	Glad, thoughtful	I am afraid that my daughter’s death is not bad enough to be spoken about.
4	Ready, excited	I had more feelings of shame and guilt than I had assumed. I try to find joy in the moments that I had with her.
5	Vulnerable, tired	I have learned that I am more ready and conscious to take Nora with me onwards.
6	Known, relieved	I want to continue writing about Nora. I feel that I have a deeper contact with her this way.

##### Fred’s Case

Fred’s poem, *My Story of Grief*, consists of 924 lines and is rich in images and memories that invite the reader to travel in time and touch upon different parts of the silver lining of suffering, love, and wisdom. Writing about the death of his daughter, Sol, he noticed all that grief amplifies: the awareness of previous miscarriages and his attitudes toward them, the longing for the second pregnancy to be alright, and coping with the tragedy of perinatal death. Fred’s journey of self-discovery not only unveils what fatherhood is about but also what the human condition through and beyond manhood can represent. He writes about a miscarriage that preceded Sol’s death years later:

*She was just under 12 weeks pregnant.*

*I say “she was pregnant” (…) because obviously the baby grows in the woman’s body.*

*It is not like the father can say “I’m pregnant,” or even “we’re pregnant.”*

*“We’re having a baby” or “my wife is expecting,” are more acceptable.*

*I feel that language is inadequate at times.*

*Doesn’t the pregnancy also belong to the father? Somehow?*

*It feels undefined.*

*Abstract.*

*A part of my soul was invested. (Fred’s poem, lines 99–109)*

This stanza gives an account of the father–child relationship that emerges during pregnancy, along with the wish of many fathers for connecting emotionally with their child during pregnancy (Bonnette & Broom, 2012). Fred also reflected the importance of continuing the bond with the deceased as part of grief integration (Klass, 2006) because it seems that his daughter, Sol, catalyzed a process several years long of reevaluation and transformation of his life purpose involving, among others, engaging himself in volunteer activities to support bereaved parents and changing careers.

Now, it is fair to say that not all of our participants wrote and shared the way Artur, Fred, or others did. It is difficult to define what dropout is about in our study because some of those who withdrew from the course attended the individual guidance sessions and/or the emotional first aid sessions with the therapists we collaborated with. What we can say is that, in similar writing courses for mothers, there was larger recruitment and retention of participants (Lehmann et al., 2022). One father wrote in the evaluation survey, I personally feel that it was ok to attend to the course but that I did not get so much help on my way forward with my grief process or how to be more aware and safer with my own feelings. (Participant 4, ES1) His feedback is important, and although his child had died because of a long-term illness and he could not attend all the sessions because of his job, it might be the case that therapeutic writing does not work for all men and/or that it is to be set as part of other interventions.

However, all three participants who responded to the evaluation survey in the second group affirmed that they would have liked to have extra sessions. We asked this group explicitly for feedback about what they thought would motivate more men to enroll in these kinds of courses. What some of them said was that “responsibilities at home” (Participant 3, ES2) could be a constraint and that “it takes time for men (generally speaking) to feel themselves safe enough to share in front of others” (Participant 1, ES2), to which another added the following:

I had been waiting for a course like this to be offered (...). I thrived best in the sessions with three participants, but this can be the introvert in me speaking. I liked a lot how the set-up was made so we could share as much or as little about what we wrote, but we could anyway speak about what we had written. (Participant 2, ES2)

This highlights that there were individual preferences about what makes low-threshold interventions helpful.

### Fourth Stanza: Grieving as Process

*How to formulate my grief? Resistance.*

*Confusion: What’s grief, what’s me?*

*Difficult to write a reflection,*

*to be in contact with feelings.*

*It turns out a little cliché, a little standard.*

*Writing another draft,*

*Wanting it to be a good one.*

*Reading out loud what’s on paper?*

*Hearing the critic’s voice within,*

*The perfectionist, the vulnerable within.*

*Difficult to get started and find words,*

*and difficult to stop writing afterwards.*

*How unique is grief!*

*In this writing group we built respect*

*for what happened to all of us.*

*It’s touching to listen to one another,*

*despite the overwhelm.*

*There are so many men who grieve in me:*

*The irritable, the impatient,*

*the enthusiastic, the egoistic,*

*The ashamed, he who struggles*

*with uncertainty.*

*There’s also a man within who steps back:*

*the one who finds energy, the one who takes perspective,*

*the one who holds an overview of life,*

*the one who keeps going, resolving,*

*the one who loves…*

*What can these teach me?*

*Looking at the bright side,*

*a provoking statement.*

*Choosing how to take things*

*pushes our boundaries.*

*Finding a sense of inner worth,*

*Learning to stand for ourselves,*

*because it is difficult, to grieve.*

*It feels easy to say it. It’s not.*

*Listening to others is humbling,*

*We mirror each other, the unexpected*

*shape that our family gained.*

*Meeting at diverse phases of life,*

*it gives perspective.*

*Compassion for others can be stronger*

*than compassion for self.*

*Interesting noticing that.*

*Gratitude.*

*Finding meaning is a practice,*

*being of support to others helps.*

*It’s a way to have peace.*

*It feels good, to shape grief with language,*

*and accept what can’t be said.*

*The book of grief*

*already has a cover sketch.*

*Holding the book close to my heart,*

*I walk onwards…*

*Grieving as Process* is a poetic representation that renders the experiences of participants with a sense of humanity that belongs to grief as an existential condition; it connects grief with important attitudes to be integrated into life through attention, compassion, and flexibility. The bereaved parents that participated in our courses found use in exploring the mind as a dialogical scene (Hermans, 2001) as they identified and elaborated upon different positionings of the self that were active as they grieved and aimed at restoring their lives after the loss. Giving room to different parts of the self as one writes about the experiences of grief has proven to be helpful in finding meaning and integrating grief to a renewed sense of identity (Den Elzen, 2021; Lengelle, 2021). Bringing the diverse positionings of the self into awareness also reflects, similarly to the dual process of grief, how life and grief are intertwined process to be balanced (Stroebe & Schut, 2010). The poem then illustrates the nuances of men’s experiences of resistance, along with how important it is for men to focus on aspects other than grief in their daily lives (Huang et al., 2023; Mota et al., 2023; Proulx et al., 2016; Zylla, 2017). Recent research findings have even suggested that an adaptive recovery in bereavement can be also achieved by focusing on more instrumental aspects, such as having a focus on living toward the future while reducing the focus on loss-oriented aspects of bereavement (Huang et al., 2023). In addition, the poem also exemplifies that a dialogical perspective isn’t limited to internal positionings of the self but is directed to a sense of otherness (Hermans, 2001; Lehmann & Brinkmann, 2019), highlighting the importance of peer support, the strong effect of understanding, and compassion from other bereaved.

#### Integrating Perspectives: The Participants’ Narratives on Grieving as Process

During the last session of each group, the participants wrote a collective poem starting with the prompt “A door is opened.” Each participant was asked to write at least one line. Once all the participants shared their suggestions in the chat, we reread them together and reorganized the lines until we had agreed that the creation was ready.

**Group 1, Autumn 2022**

*A door is opened*

*darkness, on the inside.*

*A future that never came into being.*

*Grief is here.*

*I was looking forward to getting to know you*

*You will always be in our memories,*

*you will, always, be there.*

**Group 2, Winter 2023**

*A door is opened*

*What will meet me once I go in today?*

*Fear, Shame, Guilt, Love?*

*See you there, you see me.*

*I am here,*

*you are here.*

These two poems head in a similar direction with the poetic representation about grief as a process, suggesting that the integration of grief is an ongoing practice and that the online course on therapeutic writing provides tools to help them working with emotions and strengthening the bond with their children. Some studies suggest that the less the parents are able to come to terms with the death of their children, the more likely they are to report severe intensity of symptoms in their grief (Keesee et al., 2008). At the same time, making sense of a child’s death is one of the most challenging aspects of the emotional processing and integration of grief, given the meaninglessness of this tragedy itself (Lehmann et al., 2022).

Online courses in therapeutic writing are only one of the multiple resources that bereaved parents can access, and even if writing proves to be helpful, grief and emotional awareness are an ongoing process, so the purpose of writing is not necessarily fulfilled by the end of the course but can be incorporated as a habit moving forward. As one participant added, “This course started a process, so that I now feel that I can continue doing it myself at home, using time for writing, for myself, and for her [daughter]” (Participant 3, ES1).

In Norway, at times, it is difficult to find grief support, so low-threshold interventions and contact with peer-support organizations are essential. The varied techniques and approaches in the courses were also experienced as positive because many participants shared that “I have gained insight about new techniques and aspects of previous modalities of writing that I will bring with me onward” (Participant 2, ES2) or that “I liked it that I could challenge myself, and since there were many different practices, I found some that I was more interested in, some that met my needs” (Participant 3, ES1). Excerpts such as this confirm the importance of holding a pluralistic attitude (Steffen et al., 2022) in the development of courses in therapeutic writing.

## Conclusion and Implications

Our exploratory study of the experiences of fathers attending an online course of therapeutic writing after the unexpected death of a child, here set in the context of Norway, sheds light on the emotional tonalities of men’s grief. The use of poetic representation as a form for qualitative inquiry facilitated the understanding of such tonalities; these evocative syntheses put into words layers of our participants’ experiences that they struggled to verbalize. For instance, grief nuanced the perception of temporality for most of our participants giving account of the uniqueness of emotional rhythms to process difficult emotions and memories. Only for some of them the perception of temporality was explicitly affected by the COVID-19 pandemic. In addition, resistance appeared to be a salient feature in the experiences of our participants. An advantage of such resistance could be a more explicit focus on restoration of life after the death of their children, the downside of this being avoided, and/or a limited literacy of emotions. Emotional awareness, writing skills, and communication skills are deepened and developed during the context of courses alike ours, but for fathers whose emotional avoidance or illiteracy is strong for many reasons, therapeutic writing might be difficult.

For many of our participants, writing became a helpful tool for meaning-making, affective processing, peer support, and a dialogical platform to continue a bond with their children. Given that fathers have unique experiences of grief and that the death of their children happens during specific periods of their life course, it is important to diversify the offers available for bereaved parents. Online or onsite groups of therapeutic writing might not work for all men, and within the setting of a group of therapeutic writing, it is crucial to have a pluralistic set of techniques and tools that can meet their individual needs and desires.

To the best of our knowledge, this is the first study of its kind in Norway, and there are various aspects that can be considered in further studies. First, for those men for whom the course worked, it appeared to be transformative, as documented in the current article. The emotional awareness and willingness to work with emotions, as well as having the time to write and the motivation and/or experience to do it, are not to be underestimated. Second, the role of personality, culture, and developmental trajectories should be further addressed in the data collection of similar studies in the future. Further research could target these features more explicitly to map the needs and wants of mourning fathers, as much as those of mourning mothers. Although it is important to simplify data collection for participants, it could also be ideal to have more longitudinal data from men. Having access to more of the diary excerpts of participants or recording the course sessions could serve this goal. Third, the number of participants in courses for fathers was lower than in similar courses offered to mothers (Lehmann et al., 2022). Whether it is the social representations of gender or another factor, it is important to break the stigma around men’s grief, both to motivate men to search for and accept support and to make support more available and effective for them. Developing campaigns targeting these topics could motivate more men to enroll in studies, hence lowering the accessibility barriers for support.

Fourth, further considerations about the course content, schedule, and pedagogical style could be considered. For instance, addressing the effects of these courses online and onsite could provide insights about settings that promote or disrupt working with emotions. For most of our participants, taking the course online came in handy for practical reasons. We did not address nor evaluate how the participation in an online course might have or not enhanced awareness and/or disclosure of emotions. Extending user involvement and the pilots of such courses could provide wider learning experiences to improve the course structure and contents. Fifth, given the popularity that the individual writing sessions had among our study participants, further studies could contrast the use of techniques in therapeutic writing in different online or onsite settings both on a one-to-one or group basis. Sixth, there are many other writings of the study participants that we did not include because of limits in the length of the present article or because of the privacy of our participants. Seeking permission to draw on these in further qualitative studies could enrich our understanding of therapeutic processes prompted by such writing assignments.
